# Complete rat spinal cord transection as a faithful model of spinal cord injury for translational cell transplantation

**DOI:** 10.1038/srep09640

**Published:** 2015-04-10

**Authors:** Dunja Lukovic, Victoria Moreno-Manzano, Eric Lopez-Mocholi, Francisco Javier Rodriguez-Jiménez, Pavla Jendelova, Eva Sykova, Marc Oria, Miodrag Stojkovic, Slaven Erceg

**Affiliations:** 1 Stem Cell therapies in Neurodegenerative diseases Lab, Research Center “Principe Felipe”, c/Eduardo Primo Yúfera 3, 46012, Valencia, Spain; 2 Institute of Experimental Medicine, Department of Neuroscience, Academy of Science of the Czech Republic, Prague, Czech Republic; 3 Neuronal and Tissue Regeneration Lab, Centro de Investigación Príncipe Felipe, Valencia, Spain; 4 Translational Research in Fetal Surgery for Congenital Malformations, Center for Fetal, Cellular and Molecular Therapy, Cincinnati Children's Hospital Medical Center (CCHMC); 5 Spebo Medical, Leskovac, Serbia; 6 Human Genetics Department, Faculty of Medical Sciences, University of Kragujevac, Serbia

## Abstract

Spinal cord injury (SCI) results in neural loss and consequently motor and sensory impairment below the injury. There are currently no effective therapies for the treatment of traumatic SCI in humans. Various animal models have been developed to mimic human SCI. Widely used animal models of SCI are complete or partial transection or experimental contusion and compression, with both bearing controversy as to which one more appropriately reproduces the human SCI functional consequences. Here we present in details the widely used procedure of complete spinal cord transection as a faithful animal model to investigate neural and functional repair of the damaged tissue by exogenous human transplanted cells. This injury model offers the advantage of complete damage to a spinal cord at a defined place and time, is relatively simple to standardize and is highly reproducible.

Successful clinical trials in treatment of SCI cannot be initiated without previous preclinical studies using adequate animal model that closely mimics the loss of function that occurs in humans. In the last decade diverse cell-based therapies have shown certain potential incorporating new neural cells into the milieu of a traumatic spinal cord injury. These cell-based treatments are designed to regenerate or remyelinate axons providing new oligodendrocytes or simply reconnecting injured tissue with newly generated neurons^1^,^2^,^3^,^4^,^5^,^6^,^7^,^8^. However, a proper and standard animal model of injury will allow better understanding of the biological and molecular changes along the injury and easily set up a platform to test potential therapeutic strategies. Widely used animal models of SCI include complete or partial transection or experimental contusion and compression, with both bearing controversy as to which one is more faithful to the human SCI functional and anatomical consequences. Human traumatic SCI is classified into five categories by the American Spinal Injury Association (ASIA) and the International Spinal Cord Injury Classification System, from incomplete to complete (E to A, respectively). The complete transection animal model reproduces the complete spinal cord injury in humans defined by ASIA as “no preservation of motor and/or sensory function exists more than 3 segments below the neurological level of injury”^9^. Nowadays, the human SCIs are likely to be much more complex than those experimentally provoked in rat models. Rats result to be a convenient model for spinal cord study, due to a low cost, easiness to care for, low incidence of surgical infections, and well established functional analysis techniques, although anatomical differences of the axonal tracts should be taken into account. Here we present a detailed surgical procedure of complete spinal cord transection and oligodendrocyte (OPC)-directed differentiated human embryonic stem cells (hESC) transplantation. Functional locomotion rescue of OPC transplanted group indeed support the use of this model for functional repair after severe SCI by exogenous human transplanted cells. Complete spinal cord transaction model was widely used to investigate the regenerative potential of different types of human cells: umbilical mesenchymal stem cells^10^, embryonic stem cells derived oligodendrocyte progenitors^1^, dental pulp-derived stem cells^11^ motoneuron progenitors^1^, olfactory ensheathing cells^12^,^13^, neural stem cells^5^. However, a surgery and cell transplantation protocol has not been so far sufficiently detailed to efficiently generate a reproducible and standardized model.

## Results

### Presurgical Procedure

**Figure 1:**
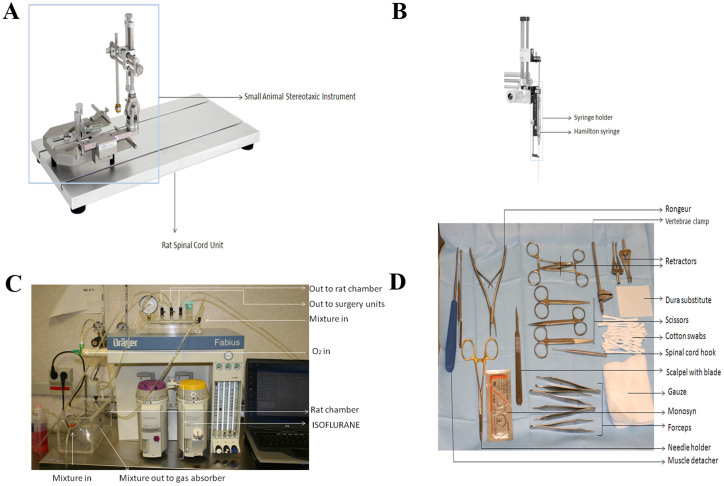
Instruments and equipment used in the protocol. (A). DKI 900 Small Animal Stereotaxic instrument mounted on DKI 980 Rat Spinal Cord Unit. (B). DKI 5000 Microinjection Unit with 5001 Holder. (C). Anesthesia Workstation (FABIUS, Dräger) connected to Plexiglas chamber and Gas absorber (CA-AG1000, Cibertec, Spain). (D). The list of surgical instruments used in the study.

All small surgical instruments were sterilized in stainless Steel Sterilization Container by autoclave. Large instruments and equipment were sterilized with 70% ethanol. The heating pad, stereotaxic instrument and Rat Spinal Cord Unit were mounted as shown on Figure 1 A–C. The small surgical instruments were additionally sterilized maintaining them in Hot bead Sterilizer. Small cuts of GORETEX (1 × 2 cm) were sterilized under the UV light and kept in flow laminar hood until use. The Anesthesia Workstation and the instruments on the surgical table are set up as shown in Figure 1 C and 1 D. For analgesia purpose, half an hour before the surgery the morphine is injected subcutaneously (2.5 mg/kg b.w.). Enrofloxacine is also administrated subcutaneously according to the animal body weight (12 mg/kg b.w.) for antibiotic prophylaxis purposes and later every 24 hours during 7 days of postoperative period. For cell transplantation from different species of the recipient immunosuppression is required and Cyclosporine A (20 mg/kg b.w.) should be injected subcutaneously daily starting two days before the transplantation until the end of experiment.

### Surgical procedure

**Figure 2:**
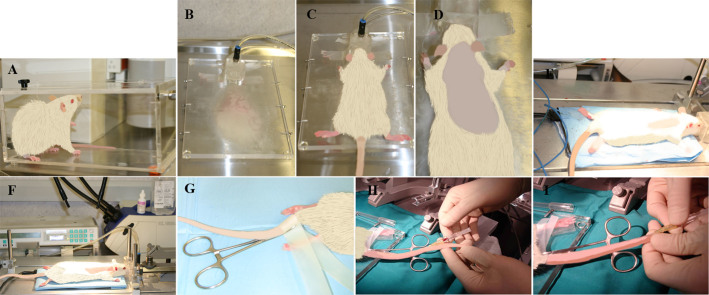
Presurgery procedure. (A). Rat in the chamber during the anesthesia. (B). Surgery unit connected with rat anesthesia mask. (C, D, E and F). Rat positioned in surgery unit with rat anesthesia mask. (G). Rat ready for introducing intravenous cannula in caudal tale venous. (H and I). Procedure of introducing intravenous cannula in caudal tale venous of the animal.

**Figure 3:**
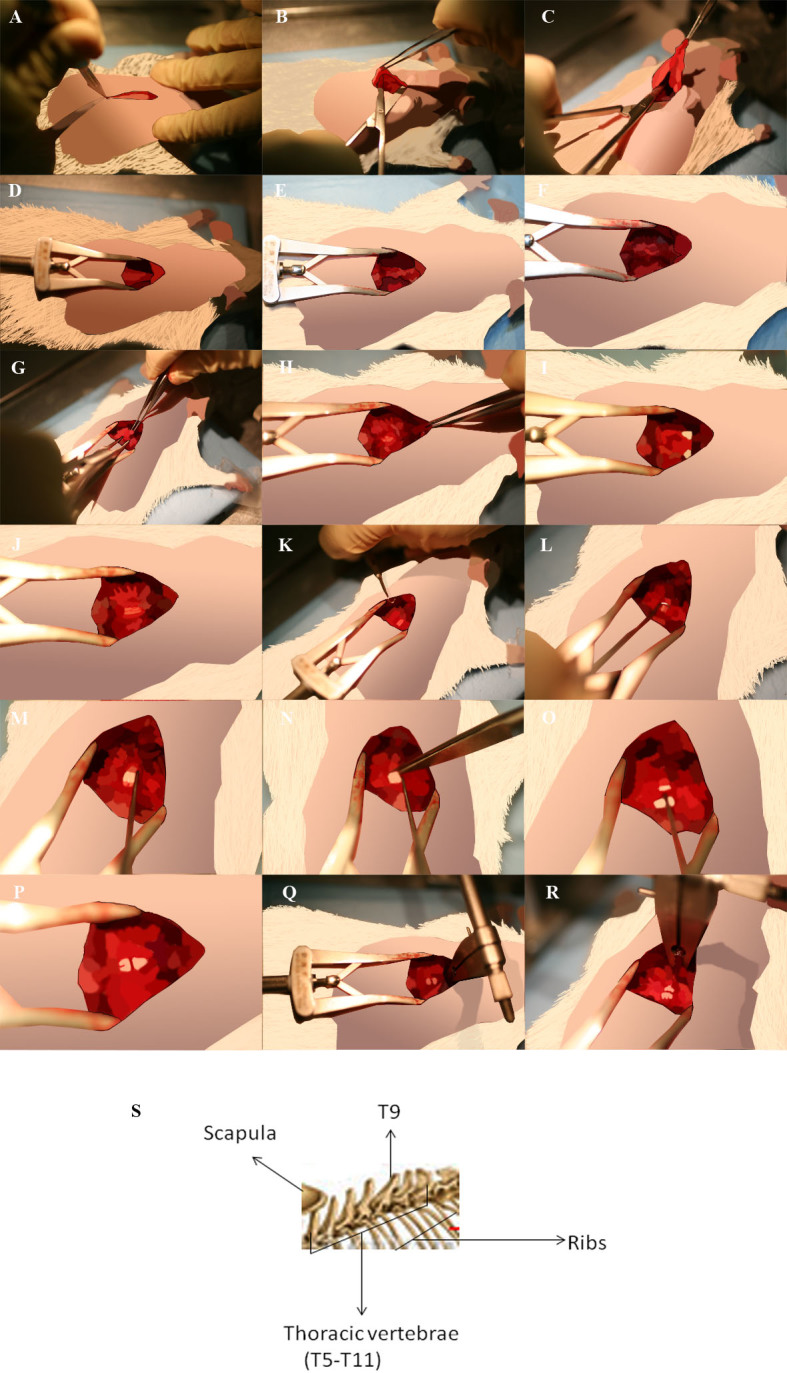
Surgery procedure. (A). A longitudinal incision of skin with scalpel blade. (B and C). Removal of fat tissue. (D). Alm retractors positioned to keep the incision widely open. (E). Exposed vertebral column T7–T10 after muscle reflection. (F). Exposed vertebral column T7–T10 after detachment of the spinotrapezium muscle from bone on the spinal laminaes. (G and H). Removal of residual connective tissue and remaining muscles by iris scissors. (I). Position of wet surgical gauze to stop eventual bleeding. J. Spinal cord at T7–T9 level after laminaectomy. (K and L). Positioning of Spinal Cord Hof. (M). Lifting the spinal cord. (N). Cross-sectional transection of the spinal cord using scissors. (O). Lifting the meninges to verify the complete transection. (P). Spinal cord cut. (Q and R). Immobilization of the spinous processes using vertebral clumps vertebral column T10. (S). Rat spine vertebrae from T5–T11. To recognize T9 it is very important to localize T10 which have different spinous processes.

**Figure 4:**
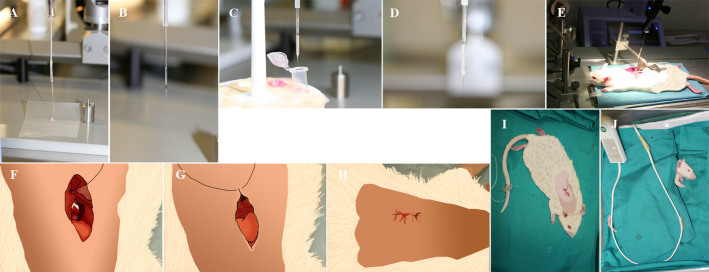
Preparation of the cells for transplantation and finishing the surgery. (A). Silicon-coated pulled 100 μm glass tip with silicon tube connected with the 50 μl Hamilton syringe. (B). The glass tip immersed in vegetal oil. (C). Immersion of glass tip into cell suspension. (D and E). Glass tip filled with cell suspension. (F). Suture of the deep and superficial muscle layers with Silkam black 3/0. (G). Suture the skin with 4/0 sterile suture. (H). Surgery completed. (I and J). Rat on heating pad.

The Anesthesia Workstation is first set up at 3% of isofluorane within 0.5% oxygen flow connected to the Plexiglas inducer chamber as it is shown in Figure 2A. Rats go into deep anesthesia plane about 1 minute after, when the muscles are relaxed. The anesthesia stage is confirmed checking the pedal, palpebral and corneal reflexes. The rat is then positioned on heating pad over the Spinal Cord Unit with stretched anterior and posterior legs (Figure 2D and 2C). The rat's head is positioned in rat anesthesia mask (Figure 2D, 2E and 2F) and the Anesthesia Workstation is set up at 2% of isofluorane. This flow is maintained until surgery is completed. Dorsal area between the neck and hindlimbs is shaved extending ~2 cm bilaterally from the spine (Figure 3A) and then disinfected with Clorhexidine digluconate 1% (It is recommended to do this procedure in the area different from the surgery zone). The intravenous catheter is introduced in caudal venous tale for fluidotherapy (Figure 2G–I). The syringe holder with the 20 ml syringe filled with physiologic saline solution 0.9%, connected to a perfusion device (Figure 2G–I), is turned on and the intravenous catheter connected to the syringe. The flow is set up at 2 ml/h and maintained throughout all surgical procedure. Eye dehydratation is prevented by applying carbomer ophthalmic gel 2% (1 drop to each eye). A longitudinal incision of approximately 2.5 cm is performed with scalpel blade (Figure 3A). After removing the fat tissue (Figure 3B and 3C) the moisture is kept with physiologic saline solution 0.9%. The muscles overlying the vertebral column are reflected exposing the vertebral column T7–T10 (Figure 3D) and the alm retractors positioned to keep the incision widely open (Figure 3D). The spinotrapezium muscle is detached from bone on the spinal laminaes using the scalpel blades or raspatory-peek handle (Figure 3E and 3F) and the connective tissue and remaining muscles are removed by iris scissors to be able to see clearly the bone structures (Figure 3G and 3H). The thoraco-dorsal arteria usually is visualized crossing throught T6 and is important to maintain it intact to avoid any hemorrage complication. Figure 3S shows a draw of the thoracic segments T5–T11. Under the headband magnifier the T9 spine segment backwards is carefully lifted while introducing slowly a Rongeur of a very fine-pointed side-cutting. First remove T9 spine and then partially remove the lateral apophasis at T9 and T8 level (Figure 3I and 3J). Dura is cut using Von Graefe Knife (Figure 3J) and 1 drop of Lidocaine solution (2%) is added directly on the lesion. The spinal cord is lifted using Spinal Cord Hook (Figure 3K, 3L and 3M) and the cross-sectional transection of the spinal cord is performed using thin scissors (Vanna Spring Scissors) (Figure 3N and 3O). This procedure frequently causes meninges to bleed, being handled with compression of the affected portion with wet surgical gauze. In order to remove the tissue between two cuts Vessel Dilating Probe needs to be passed through meninges tissue (Figure 3P). It is very important to cut any residual fibers and to verify complete transection. It has been reported that 5–10% of spared white matter in the ventrolateral funiculi is sufficient for sustained rat locomotion^14^,^15^. The spinous processes are immobilized using vertebral clamps fixed to the Spinal Cord Unit securing the T10 vertebral segment (Figure 3Q and 3R). Cells, prepared as described in Methods are positioned into the glass pipette fixed to Hamilton syringe which is adapted to a microinjector at the stereotaxic unit over the spinal cord surface; 3 mm (caudally) bellow the lesion as is shown on Figure 4E. The glass pipette is lowered 1 mm and a total of 5 μl of cell suspension is delivered at 2 μl/min. An automatic microinjector is also recommended like a Nanomite, Infuse/Withdraw (70-3601, Harvard Apparatus, USA). Excesive rate of injection leads to the grafting decrease. The glass pipette is left in the injection site for 2 minutes without injection to avoid leaking of the injected cells. Last steps are repeated by injecting the glass pipette in the second and third caudal position as well as in the equivalent three positions rostrally, 3 mm above the lesion. The injections in host spinal cord cranial and caudal to the lesion epicenter were performed in order to avoid the cavitations epicenter, hemorrhagic necrosis, and inflammation, which might decrease cell survival and integration. The glass pipette is removed carefully from the injection site. Vertebrae clamps and retractors are removed. The laminectomy was covered with GORETEX synthetic dura (Figure 4E). The deep and superficial muscle layers and the skin are carefully sutured with Monosyn violet 4/0 (Figure 4F, 4G and 4H). The animal is then recovered of anesthesia on a heating pad until alert and mobile (Figure 4I and 4J) and the bladder is manually pressed until completely empty.

### Cell preparation for transplantation

For transplantation the cells are disaggregated mechanically with a glass pipette and centrifuged for 2 min at 50 *g*, room temperature. The cells are disaggregated by pipetting to single cell suspension in culture medium. Immediately before transplantation, the cell viability is checked by trypan blue and cell populations with >95% viability are used for transplantation. The cell solution is prepared at 100.000 cells/μl and 1,6 million cells injected per animal. The silicon-coated 100 μm glass tip with silicon tube connected to the 50 μl Hamilton syringe needle and microinjector are mounted on stereotaxic frame (Figure 4A). Using a stereotaxic manipulator arm and injection unit the glass tip are immersed in vegetal oil (Figure 4A). The role of the oil is to push uniformly the cell sample. Approx. 20 μl of vegetal oil is aspirated in the glass pipette using microinjection unit. Using a stereotaxic manipulator arm and injection unit (Figure 4B) 16 μl of cell suspension is aspirated in the glass pipette using microinjection unit (Figure 4C and 4D).

## Results Supporting the Procedure

**Figure 5:**
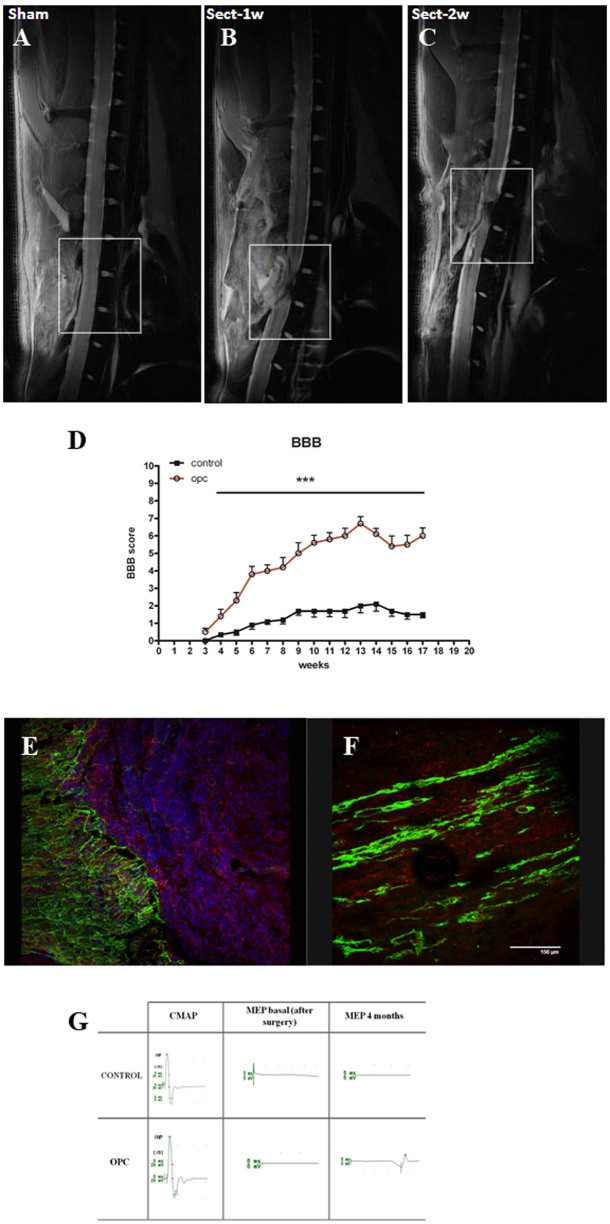
(A–C) NMR of the rat with and without complete transaction. (A). Sham. (B). Injured rats (laminaectomy with complete transaction of spinal cord) 1 week post surgery. (C). Injured rats (laminaectomy with complete transaction of spinal cord) after 2 weeks post surgery. (D) BBB score during 4 month postsurgery. Starting 4 weeks after the transplantation, a significant increase (P < 0.001) in locomotor recovery, determined by the BBB locomotor rating scale, was observed in OPC transplanted animals compared to controls. The values are presented as mean ± s.e.m. (E, F) Immunohistological analysis of completely transected spinal cord with and without cell treatment. (E). Spinal cord (Control) 4 months after complete transaction. No signs of axonal regrowth of existing neurons (green-NF200). GFAP (red), DAPI-blue. (F). Spinal cord 4 months after complete transection and OPC treatment. NF70^+^ cells reconnecting the lesion site and coinciding with improvement of locomotor activity. (G) In vivo electrophysiology. Representative electrophysiological recordings in control and transplanted animals (OPC) during 4 months of the experiments. The basal motor evoked potential (MEP) is the same in both cases after the complete transection of spinal cord. MEP was registered in transplanted animals after 1 month and was maintained until the end of experiment. MEP was not registered in control animals. CMAP- compound motor action potential.

Nuclear magnetic resonance (NMR) imaging provides a noninvasive method for studying the integrity of spinal cord and in the case of spinal cord injury faithful tool to follow the spinal cord damage after spinal cord transection *in vivo*. NMR images showed a clear and persistent lesion with no spared axons in the lesion site as shown in Figure 5B and 5C. Regenerative effects of OPC cells in rat's transected spinal cord are already described with more details^1^. Locomotor tests such as open field locomotor scale, described by Basso, Beattie and Bresnahan (BBB) are used to assess locomotor recovery after complete transaction injuries in rat spinal cord^16^ with and without transplanted cells. In this locomotor assay rats are trained weekly to move in an open field which is a molded-plastic circular enclosure with a smooth, nonslip floor. Rats were allowed to move freely and are scored during 4 minutes for their ability to use their hindlimbs. Joint movements, paw placement, weight support, and fore/hindlimb coordination are judged according to the 21-point BBB locomotion scale. Before the injury, all animals showed normal locomotor activity, scored as 21 on the BBB scale, although all injured rats manifested complete hind limb paralysis 7 days after injury, resulting in a score of 0. The BBB scores were in the range of 0–1 or 2 in the control animals during the 4 months after SCI (Figure 5D). In contrast animal group transplanted with OPC showed hind limb functional locomotor recovery which increased gradually after 3 weeks of transplantation. Four months after transplantation OPC transplanted animals displayed BBB scores significantly (P < 0.001) higher than that achieved by the control group reaching a final average BBB score of 6 (Figure 5D).

The degree and functional significance of complete transection in the host tissue is evaluated by immunohistological techniques. Immunohistochemistry analysis using the antibody against Neurofilament 200^+^ fibers has been shown that area of surviving white matter in cross-section through the center of lesion site has disappeared or has significantly reduced compared with intact animals, indicating the absence of spontaneous axonal regeneration in non-transplanted rats after complete transection (Figure 5E). Immunohistochemistry analysis confirmed previous findings that regenerative effect after transplantation is due to transplanted cells differentiated to neurons which coincides with locomotor activity^1^. The presence of neurons of human origin in the lesion site is confirmed by immunoreactivity against NF70, human specific marker (Figure 5F).

Another method of reconnection of damaged tissueily is assessing motor pathways is the simultaneous stimulation of the motor cortex using transcranial magnetic stimulation (TMS) and voluntarily contraction in a target muscle as a non-invasive, painless and safe method in assessment of human central and peripheral motor pathways^17^,^18^,^19^. The lower limb motor evoked potentials (MEP) determine the severity of spinal motor damage. Complete transection of spinal cord produces flat MEP after surgery, without recovery after 4 months (Figure 5G). Our results have shown that OPC cells transplantation immediately after surgery induces MEP after 1 month which is maintained for another 4 months, clearly indicating the regenerative effects of these cells^1^ (Figure 5G).

## Discussion

Our results confirm that rat model of complete transection is reproducible and simple to standardize model for SCI, faithfully mimicking the most severe clinical cases of SCI in humans. The most important advantage of this model is completeness of the injury that can be performed at defined time and place. As there are no spared axons in the lesion site, with this model is faithful for interventions, pharmacological or cellular, designed to promote axonal regeneration or reconnection using exogenous cell source^1^,^20^,^21^. This kind of surgery enables to follow the behavior of lesion site in a more precise manner due to the fact that the damage to the nerve fibers is not spread out like in other models^22^ as it is shown by NMR in our study. Although weight drop or contusion SCI models offer the possibility to generate different degrees of injury severity and functional outcomes, many groups showed spontaneous recovery and locomotor improvement in moderate injuries^23^. These consequences can mask the potentially beneficial effects of exogenous strategies such as cell therapy and therefore hinder this kind of treatment. The major flaw of complete section is high severity of the injury reflected in slightly elevated mortality rate during the post-trasplantation period comparing to other models with the advantage that any regenerative effect can be attributed exclusively to exogenous treatments. This model together with the hemisection is also very useful to test various “bridging gap” biomaterial or device studies alone or in combination with cell grafts^24^,^25^,^26^,^27^,^28^,^29^. Various studies used this model to study the effects of combination of different scaffolds and biomaterials and stem cells on axon regeneration after injury, such as a polymer scaffold with rat bone marrow stem cells^30^,^31^. Enhancing axonal regrowth by modulation of astrogliosis by transplanted cells after spinal cord transection appears to be a promising therapeutic approach to repair the injured spinal cord^31^.

The evaluation of the functional motor recovery by BBB test reflects the regeneration of lesioned spinal cord due to transplanted cells excluding the effect of spared axons. Many studies confirm that rats with completely transected spinal cord lose their locomotor function immediately after surgery, from normal locomotion (score 21) to complete paralysis of hind limbs (score 0), with slight improvement (not exceeding score 1 or 2) during 4 or 8 months of observation^1^,^11^,^32^. The behaviour of control animals is consistent with the results obtained using human umbilical mesenchymal stem cells^10^ or olfactory ensheathing cells^13^ revealing the reproducibility of this test in complete spinal cord transection model. The advantage of complete transection of spinal cord is the fact that during the surgery procedure all residual fibers are cut. In the case of other SCI models, such as contusion model^23^ or balloon-induced spinal cord compression^22^ lesion, it is difficult to determine the contribution of spare axons to the regeneration of damaged tissue.

In conclusion, complete transection animal model of SCI causes severe behavioral (locomotor) and histological (axonal damage) changes, and has proved both useful and reliable for evaluation of rodents using different cell or pharmacological strategies^1^,^10^,^11^,^12^,^13^,^33^,^34^,^35^.

## Methods

All material and reagents as well as detailed procedure of animal care after the lesion and transplantation were listed and described in Supplementary Methods.

### Experimental individuals

Adult female rats, 2 months old, 200 g of body weight. Food and water provided ad libitum during the entire experiment. All surgical procedure steps have to be performed according to ethical procedures for the use of animals in laboratory experiments. The experimental protocol used here was approved by the Animal Care Committee of the Research Institute Principe Felipe (Valencia, Spain) in accordance with the National Guide to the Care and Use of Experimental Animals (Real Decreto 1201/2005).

#### Post-surgery procedure

Post surgery procedure are provided in Supplementary Methods.

### Cell culture and differentiation

Primary hESC colonies (H9-GFP, WiCell Inc., Madison, WI) are cultured on mitomycin C inactivated commercially available human foreskin fibroblasts (American Type Culture Collection, Manassas, VA, USA), in ES medium containing Knockout-DMEM (Invitrogen), 100 μM ß-mercaptoethanol (Sigma), 1 mM L-glutamine (Invitrogen), 100 mM nonessential amino acids, 20% serum replacement (SR; Invitrogen), 1% penicillin-streptomycin (Invitrogen), and 8 ng/ml basic fibroblast growth factor (bFGF; Invitrogen). ESC medium is changed every other day. Human ESC are passaged by incubation in 1 mg/ml collagenase IV (animal-free, Invitrogen) for 5–8 minutes at 37°C or mechanically dissected and moved to freshly prepared feeder cells.

Cells are differentiated toward OPC according to already published protocols^2^,^36^. Briefly, cell clumps are placed for 2 days in 50% hESC growth media and 50% glial restriction media (GRM)^2^ in ultra-low attachment 6-well plates (Corning). This medium is then replaced with 100% GRM supplemented with 20 ng/ml EGF (Sigma-Aldrich) and 10 μM/ml all-trans-retinoic acid (RA) for additional 7 days. During 25 days the cells are exposed to GRM supplemented with 20 ng/ml EGF. Then, the floating yellow spheres are plated on 6-well plates (BD) coated with Matrigel (1:30) for 1 week. The progenitors are migrated from the spheres and are replated, for 1 week in GRM supplemented with 20 ng/ml EGF with the same coating. At day 48 the cells are ready for transplantation.

### Behavioral testing (open field locomotor scale)

Functional recovery is assessed by evaluators blinded to treatment groups. Open field locomotor test using the Basso-Beattie-Bresnahan (BBB) Locomotor Rating Scale^14^ is performed in a plastic tray (50 × 80 × 40). One week before injury, each animal is acclimated to the open-field and scored. The BBB test is performed every week after injury during 4 months when two independent examiners observed and recorded with video digital camera (Sony) the hindlimb movement of the rats, which range from 0 (no hind movement) to 21 (normal gait). The videos are analyzed frame by frame using ImageMixer 3SE software and scored independently by two observers blinded to the treatment group.

### Electrophysiology measurements *in vivo*

The motor potentials are evoked and recorded according to the prior study^37^. The main difference in our procedure is that the cranial screw is not implanted and a needle electrode is used. According to the anaesthetics study of Oria et al.^38^ the propofol is administered intravenously as a bolus dose of 10 mg/kg. For the recording of evoked potential [MEP and compound motor action potential (CMAP)] one needle electrode is placed in the tibialis anterior muscle (cathode) and another one subcutaneously at the foot pad level (anode). For the induction of CMAP following peripheral nerve stimulation, one electrode was placed in the muscle (cathode) and another subcutaneously (anode), both near the sciatic nerve. For the induction of MEP (after central stimulation) one needle electrode was placed subcutaneously at the level of the lower jaw (anode) and a needle electrode (cranial level) was used for the cathode. For ground, an electrode was placed subcutaneously in the lumbar region. The electrophysiological recordings are performed with an electromyographer (Medtronic Keypoint Portable, Denmark) and the bandpass used is 2 Hz to 10 KHz. Throughout the experiments, the duration of the pulse is 0.1 ms. The recordings are started by measuring the maximum amplitude of the CMAP. This is achieved by stimulating the sciatic nerve with a single pulse of supramaximal intensity. In order to induce MEP, a stimulation of 25 mA intensity is applied at the needle electrode (cranial level).

### Magnetic resonance experiments

In vivo 1H-Magnetic resonance studies were performed at the NMR facility (SeRMN) of the Autonomous University of Barcelona in a 7 Tesla horizontal magnet (BioSpec 70/30, Bruker BioSpin, Ettlingen, Germany) equipped with actively shielded gradients (B-GA20S) using a quadrature 72 mm inner diameter volume resonator. Imaging parameters for these images were: effective echo time (TEeff) = 36 ms, repetition time (TR) = 2 s, echo train length (ETL) = 8, field of view (FOV) = 7 × 4 cm^2^, matrix size (MTX) = 128 × 128, slice thickness (ST) = 1.5 mm, and number of averages = 4. Using these scout images a high resolution T2-weigthed respiration gated image was acquired in the sagital plane through the center of the spinal cord with the following parameter: effective echo time (TEeff) = 45 ms, repetition time (TR) = 2 s, echo train length (ETL) = 4, field of view (FOV) = 6 × 4 cm^2^, matrix size (MTX) = 512 × 256, and slice thickness (ST) = 1.5 mm, number of averages = 8.

### Immunofluorescence

For immunohistochemistry analysis, animals were kept alive 4 months after cell transplantation. Then the animals were transcardially perfused under pentobarbital anaesthesia (80 mg/kg b.w. intraperitoneally) and fentanyl analgesia (0.05 mg/kg b.w ip) with a 0.9% saline solution followed by 4% paraformaldehyde (PFA; 158127, Sigma) in phosphate buffered saline (PBS). The tissue was included in 30% sucrose (S84097, Sigma) during two days before inclusion in Tissue-Teck OCT (Sakura Finetek U.S.A). Sagittal cryosections of 10 μm thickness were used for immunoassays.

Cryosectioned tissues were additionally fixed with 4% paraformaldehyde at room temperature for 10 min. After permeabilization with 0.5% Triton X-100 (X100, Sigma) 100% solution containing 2% goat serum (blocking solution; G6767, Sigma), the primary antibodies, mouse anti-NF200 (1:250) and rabbit anti-cow GFAP (1:500; DakoCytomation, Glostrup, Denmark), were incubated overnight at 4°C, diluted 1:200 in blocking solution. After being rinsed three times with PBS, the cells were incubated with Oregon Green-Alexa488 dye conjugated goat anti-mouse IgG or Alexa555 goat anti-rabbit IgG 1:400 (Invitrogen, CA, USA) secondary antibodies for 1 h at room temperature. All cells were counterstained by incubation with 4,6-diamidino-2-phenylindole dihydrochloride (DAPI) from Molecular Probes (Invitrogen, USA) for 3 min at room temperature followed by washing steps. Signals were visualized by Confocal Microscopy (Leica, Germany).

### Statistical methods

BBB scores is analyzed by repeated measures 2way ANOVA with Bonferroni multiple comparison test at each time point. The differences were significant when P < 0.05.

## Supplementary Material

Supplementary Information Supplementary Information
