# Supplementary material for: Complete rat spinal cord transection as a faithful model of spinal cord injury for translational cell transplantation
Source: Sci Rep. 2015 Apr 10;5:9640. doi: 10.1038/srep09640 (PMC5381701; doi:10.1038/srep09640)
Supplement: Supplementary Information [file srep09640-s1.doc]

**Complete rat spinal cord transection as a faithful model of spinal cord injury for translational cell transplantation**

Dunja Lukovic^1§^, Victoria Moreno-Manzano^3§^, Ana Alastrue-Agudo^3^, Francisco Javier Rodriguez-Jiménez^3^, Marc Oria^4^, Miodrag Stojkovic^5,6^, Martin Klabusay^7^ and Slaven Erceg^1,2^*

**Supplementary Methods.**

**Material**

***Equipment:***

- DKI 900 Small Animal Stereotaxic Instrument mounted on DKI 980 Rat Spinal Unit (David Kopf Instruments) **Figure 1A**
- Heating Pad (Ref. TCAT-2LV Physitemp)
- DKI 5000 Microinjection Unit (incl. 5001 Holder (David Kopf Instruments)
- DKI 5002 5/10 μl Hamilton Syringe Holder (David Kopf Instruments)
- Rat Vertebrae Clamp (David Kopf Instruments, **986B**)
- 50 µl Hamilton syringe (Hamilton, Reno, NV)
- Glass customized siliconized pipettes (100 µm diameter, Eppendorf)
- Anesthesia Workstation (FABIUS, Dräger) (**Figure 1 A and 1C**)
- Gas absorber (CA-AG1000, Cibertec, Spain) **Figure 1 C**
- Infusion Pump (Perfusor fm. bBraun Vetcare).
- Stainless Steel Sterilization Container (20890-51. FST)
- Hot bead Sterilizer (18000-45. FST)
- Headband Magnifier (28030-02. FST)
- Headband Magnifier (28030-02. FST)
- Scalpel Handle (10003-12. FST)
- Blades (10011-00. FST)
- Von Graefe Knife (10059-13. FST)
- Rongeur: Fridman-Person (16220-14. FST)
- Vanna spring scissors (15018-10. FST)
- Fine scissors (14094-11. FST)
- Dumont fine forceps (11254-20. FST)
- Graefe Forceps (11650-10. FST)
- Adson Forceps - DeBakey Pattern (1617-12. FST)
- Gilies Forceps (11028-15. FST)
- Baby Mixter Hemostats (13013-14. FST)
- Alm retractor (17008-07. FST)
- Raspatory Peek-Handle (FK317R. Aesculap)
- Spinal Cord Hook (10162-12. FST)
- Dura Substitute ( 1PDX-MVP300, GORE® PRECLUDE®)
- Needle Holder: (12502-12. FST)
- Monosyn violet 4/0 HR26 (G0022024, Braun)
- Intravenous Catheter (4253302, bBraun Vetcare)
- Cotton swabs
- Gauze
- Screwdriver set (30090-06. FST)
- Vessel Dilating Probe (10160-13. FST)
- Double lane Treadmill (LE8706. Panlab)

***Reagents***

- Morphine Hydrochloride 2 % (656758 bBraun Vetcare)
- Cyclosporine A 250 mg(Novartis Pharmaceuticals)
- Enrofloxacine 2.5 % (Bayer Healthcare)
- Carbomer Oftalmic Gel 2 %
- Isofluorane (469860, bBraun Vetcare)
- Lidocaine 2 % (bBraun Medical)
- Buprenorphine 0.3 mg/ml (Schering Plough, S.A.)
- Pentobarbital 20 % (Ref: Q-7090-032. Vetoquinol)
- Fentanil (Kern Pharma)

***Animal care***

Bladders were manually expressed twice daily until vesicle reflex or bladder function is restored within 2-3 weeks post-injury. The rats are inspected for weight loss, dehydration, discomfort, and autophagia.

The animals are treated with Enrofloxacine (5mg/kg b.w, sbc) for 7 days to prevent possible bacterial infections and with Buprenorphine (0.1 mg/kg b.w, sbc) for 4 days for analgesic purpose. The body weight is daily monitored and rats whose weight has declined more than 20 % will be excluded (and not represented in the experimental numbers).

One week after surgery, the animals are subjected to daily rehabilitation procedures, consisting of passive mobilization through a full range of movements to maintain joint flexibility and assessment of reflexes in the hindlimbs i.e., withdrawal reflex, toe spread response and 15 min-massage of hindlimbs. In addition to passive mobilization, the daily active rehabilitation includes daily mobilization on a treadmill (Panlab), with a rolling belt with adjustable speed (up to 150 cm/s) and swimming in a pool. Prior to surgery, all animals will be trained and exposed to the swimming pool and treadmill for several minutes per day at least during 5 consecutive days. During this time the research personnel responsible for swimming and testing handled the animals on a daily basis and fed them sweetened cereal as treats. Rats are natural swimmers and are quickly acclimatized to the swimming sessions. A swim test is performed in a rectangular Plexiglas basin (70 cm long, 45 cm wide and 25 cm deep) filled with temperate water (37ºC). Intact animals swim by paddling with their hindlimbs and the tail, holding their forelimbs immobile under the chin [^16^](#_ENREF_16).

After each pass, animals are removed from the end of the tank and given a short rest, the length of which depends on their past performance. These procedures facilitate a straight swimming trajectory across the tank and prevented escape behaviours. In addition, rats receive intermittent positive reinforcement for successful trials (sugared cereal). Initially after SCI, rats require a vest with narrow strips of closed-cell foam on the back to assist with flotation, and they primarily used the forelimbs to swim. The foam is sufficient to keep the head above the water only when the rats swam. The foam is removed after 4-5 sessions when swimming movements of the hind limbs begin to emerge and are sufficient to keep the rat afloat. Daily notation of hindlimb swim performance includes extent of movements and relative frequency of kick cycles. Time engaged in swimming is recorded for each animal to measure training effects. Rest periods of up 2 minutes after each bout of swimming are provided early after SCI to avoid inducing marked stress and non-compliance of the rat. Rest times were gradually shortened to 1 min as the hindlimb swimming movements increased.
